# Mapping nonlocal relationships between metadata and network structure with metadata-dependent encoding of random walks

**DOI:** 10.1126/sciadv.abn7558

**Published:** 2022-10-28

**Authors:** Aleix Bassolas, Anton Holmgren, Antoine Marot, Martin Rosvall, Vincenzo Nicosia

**Affiliations:** ^1^ School of Mathematical Sciences, Queen Mary University of London, London E1 4NS, UK.; ^2^ Departament d’Enginyeria Informatica i Matematiques, Universitat Rovira i Virgili, 43007 Tarragona, Spain.; ^3^ Instituto de Física Interdisciplinar y Sistemas Complejos IFISC (CSIC-UIB), Campus UIB, 07122 Palma de Mallorca, Spain.; ^4^ Integrated Science Lab, Department of Physics, Umeå University, SE-901 87 Umeå, Sweden.; ^5^ RTE Réseau de Transport d’Electricité, Paris, France.

## Abstract

Integrating structural information and metadata, such as gender, social status, or interests, enriches networks and enables a better understanding of the large-scale structure of complex systems. However, existing approaches to augment networks with metadata for community detection only consider immediately adjacent nodes and cannot exploit the nonlocal relationships between metadata and large-scale network structure present in many spatial and social systems. Here, we develop a flow-based community detection framework based on the map equation that integrates network information and metadata of distant nodes and reveals more complex relationships. We analyze social and spatial networks and find that our methodology can detect functional metadata-informed communities distinct from those derived solely from network information or metadata. For example, in a mobility network of London, we identify communities that reflect the heterogeneity of income distribution, and in a European power grid network, we identify communities that capture relationships between geography and energy prices beyond country borders.

## Teaser

Blending network communities with node metadata using compression of random walks reveals their distant relationships.

## INTRODUCTION

The network structure of a complex system provides meaningful insights into its function, dynamics, and evolution (*1*–*3*). For example, partitioning networks into internally densely connected communities or modules of nodes helps researchers understand how systems organize at different scales (*4*–*6*). However, focusing only on the network topology disregards potentially available metadata, link types, or node labels that can enrich the plain network and provide valuable information about its large-scale organization (*7*, *8*).

Researchers have used such metadata to predict missing links in real-world networks (*9*, *10*) and to better characterize dynamics and polarization (*11*). Encoding link-related metadata with multilayer networks has also proven effective for understanding various processes in systems with diverse relationships (*12*–*14*). A promising research direction is to integrate metadata in community detection, the art of finding important mesoscale structures in networks that can guide further research into understanding the functioning of a system.

Different techniques to include exogenous information in community detection methods have been recently explored (*15*, *16*) to account for node-related metadata and to improve the quality and meaning of partitions based exclusively on node-to-node relationships (*17*–*19*). For instance, extended stochastic block models and flow-based methods can exploit metadata to overcome the detectability limit when local correlations between network communities and metadata are present: Combining network structure and metadata enables more accurate community detection when densely connected nodes share similar metadata (*17*).

However, when nodes with similar metadata are far apart in the network, such that no local correlations between node metadata and network structure exist, the presence of metadata adds no value to the extended stochastic block models (*16*, *17*). Similarly, encoding metadata in flow-based modules without local correlations with the network structure, using the so-called content map equation, further divides the structural communities without revealing nonlocal relationships between node metadata and network communities (*18*, *19*). While these methods have valid use cases, they cannot additively combine network structure and metadata to highlight either the network structure or the metadata or any blending of the two, because they cannot exploit nonlocal relationships between them.

**Fig. 1.**
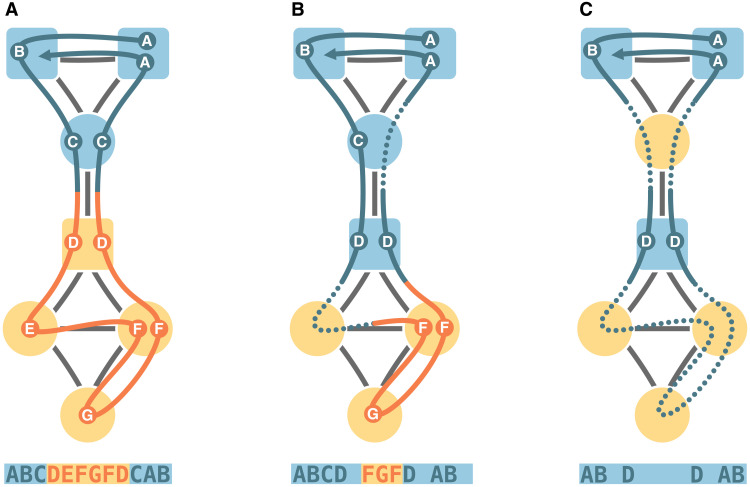
Random walks with metadata-dependent encoding probabilities. In this schematic example with single, long random walks, we encode the random walks’ next step if the target node’s metadata are the same as for the previously encoded node. When the metadata differ, encoding with a probability between 33 and 100% gives the solution in (**A**), between 10 and 33% gives the solution in (**B**), and less than 10% gives the solution in (**C**). Node shapes represent metadata, and node colors represent optimal partitions. The random walks are colored by the currently encoded module and labeled with circles when they encode a transition and with dotted lines when they skip nodes. The alphabetic codes represent the walks with this metadata-dependent encoding scheme.

To explore the mesoscopic structure of nonlocal relations between structural information and node metadata, we take a flow-based approach relying on the map equation but distinct from the content map equation. The map equation casts community detection into a compression problem by estimating the most efficient modular encoding scheme to represent random walk transitions between nodes in communities. The standard map equation encodes every step of a random walk, and the content map equation uses different codes for network transitions and metadata. Instead, we incorporate metadata by letting the encoding probability depend on the metadata of the next step’s target node and the previously encoded node (Fig. 1). This metadata-dependent encoding scheme provides a natural way to integrate possible nonlocal relationships between structural graph properties and metadata. By linking the random walk’s encoding probability to the metadata, our metadata-dependent encoding framework allows us to continuously tune the relative importance of network structure and metadata and to incorporate field-specific knowledge in the network analysis. For example, in synthetic graphs without local correlations between structure and metadata, our framework merges distant nodes with matching metadata depending on the mesoscale properties of the graph.

We show that modular compression of random walks with metadata-dependent encoding probabilities on various real-world networks reveals a variety of functional metadata-informed communities. We find that many social and spatial systems allow metadata-enriched partitions that differ substantially from those obtained from either structural network information or metadata clustering alone. For example, analyzing the spatial network of energy prices across Europe reveals regions that do not map directly to countries or price ranges but correspond instead to transnational areas characterized by socioeconomic similarities.

## MATERIALS AND METHODS

Standard flow-based community detection methods, such as Markov stability and the map equation, capitalize on random walks’ propensity to remain trapped for relatively long times in densely connected subgraphs. We take advantage of a multiscale extension of the map equation that modifies the standard Markov process with one-step transitions at Markov time 1 to shorter or longer Markov times (*20*, *21*). Changing the Markov time corresponds to changing the rate at which the transitions of the random walk are encoded. For Markov times shorter than 1, the map equation encodes the random walk more frequently than once per step, leading to smaller communities. For Markov times larger than 1, the random walk can make more than one step before the map equation encodes its transition from the previously encoded node, leading to fewer, larger communities with larger diameter. To explore relationships between network structure and metadata of distant nodes, we encode the random walker’s transition to a node as a function of its metadata and the previously encoded node’s metadata (Fig. 1).

### Modeling random walks with metadata-dependent encoding probabilities

We consider a connected and possibly weighted graph *G* = (*V*, *L*) with *N* = ∣*V*∣ nodes and *K* = ∣*L*∣ edges. For simplicity, we assume that the graph *G* is undirected, but a similar reasoning holds for primitive directed graphs as well. Assuming that nodes are associated with some categorical, scalar, or vectorial metadata, such as gender, occupation, or income, there exists a function *f* : *V* ⟶ S that maps each node *i* to an element *f_i_* of the generic set S, where S ⊆ ℕ for categorical data and S ⊆ ℝ*^d^* for scalar or vectorial data. As shown recently, the symbolic dynamics *F*({*i*_0_, *i*_1_, …, *i_t_*, …}) = {*f*_*i*_0__, *f*_*i*_1__, …, *f_i_t__*, …} associated with the generic trajectory {*i*_0_, *i*_1_, …, *i_t_*, …} of an unbiased discrete time random walk on *G* starting from node *i*_0_ retain plentiful information about the underlying distribution of metadata at different scales (*22*, *23*).

In the absence of metadata, the symbolic dynamics *F* are trivial. However, the underlying random walk statistics depend on the structure of *G*, including its degree distribution, degree-degree correlations, the presence of clustering, communities, and so forth. Several flow-based community detection algorithms exploit this connection between structure and dynamics. Conversely, if the graph *G* presents no structural heterogeneity, such as in an infinite lattice or a regular random graph, then the statistics of the symbolic dynamics *F* depend only on the presence of metadata correlations, since spurious effects due to local trapping are averaged out in walks of infinite length. We interpolate between these two extremes by considering a random walk with metadata-dependent encoding probability: The random walk can skip encoding nodes if their metadata differ from the metadata of the previously encoded node. The corresponding metadata-informed communities contain nodes that appear in relatively long uninterrupted encoding sequences (Fig. 1). For straightforward community detection, we assemble the probability flows of sequential encodings in a metadata-dependent encoding graph where all probability flows sum to one and the metadata-informed communities form dense subgraphs.

**Fig. 2.**
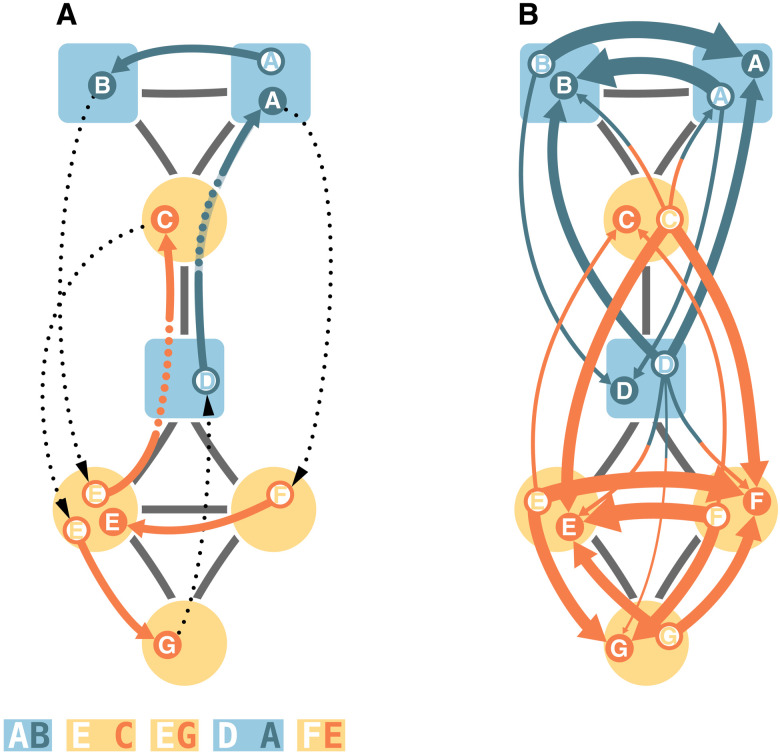
Generating the metadata-dependent encoding graph. (**A**) For ergodic results, we consider random walks that restart after each encoding at a random node proportional to its stationary visit rate. The random walk is colored by the currently encoded module and labeled with circles when it encodes a transition, with dotted colored lines when it skips nodes and with thin dotted lines when it restarts. If the target node’s metadata are the same as for the restart node, then we always encode. When the metadata differ, we encode with a probability of 5% like in Fig. 1C. The alphabetic codes represent the walk with this metadata-dependent encoding scheme. (**B**) Aggregating all encoded transitions and dividing by the total number of transitions give the metadata-dependent encoding graph. The line widths represent the probability flows of next encodings. For visual simplicity, we show only probability flows above 1%. Node shapes represent metadata, and node colors represent optimal partitions.

Constructing the metadata-dependent encoding graph from a single, long random walk that continues after each encoding, as illustrated in Fig. 1, can produce unreliable results that depend on the starting node. For ergodic results, we consider random walks that restart after each encoding at a node *i* proportional to *i*’s stationary visit rate *p_i_* of a standard random walk without metadata-dependent encoding. The fragmented random walk starts on node *i*, steps to node *j*, and encodes the transition from node *i* to node *j* with probability ε*_ij_*, where ε*_ij_* is some meaningful function of the metadata *f_i_* and *f_j_*. With probability 1 − ε*_ij_*, the random walk skips encoding, continues to node *k*, encodes the transition from node *i* to node *k* with probability ε*_ik_*, and so on. After a transition is encoded, the random walk restarts at a random node proportional to its stationary visit rate, and the procedure repeats (Fig. 2A). When restarting after each encoding, communities with long encoding sequences become communities that frequently contain encoded transitions from the restart node to the next encoded node. Aggregating all encoded transitions and dividing by the total number of such transitions give the metadata-dependent encoding graph (Fig. 2B). Without encoded restarts, the metadata-dependent encoding graph takes the same form as if we generated it by a single, long walk, save for possibly nonmatching in- and outflow volumes at nodes. Because exiting communities with a low probability and remaining trapped in communities for extended times are equivalent to our modular compression approach, we retain the interpretation that communities contain nodes that appear in relatively long uninterrupted encoding sequences.

The distribution of encoding probabilities represents the traversal resistance induced by nodes on walkers depending on their origin and defines the walks’ horizon. In the simple case ε*_ij_* = 1, any walker starting at node *i* will encode as soon as it reaches node *j*. Node *j* presents an infinite resistance to all walkers originating at *i*. If ε*_ij_* ≃ 0 instead, then typically none of the walkers starting at *i* would ever encode at *j*. We can drive the walkers starting at *i* toward nodes with specific metadata, by letting ε*_ij_* depend on the metadata *f_i_* and *f_j_*. In particular, if ε*_ij_* = δ_*f_i_*,*f_j_*_, then walkers from node *i* will only encode at nodes whose metadata are identical to those of *i*, irrespective of their distance on the graph *G*. All encoded transitions would occur between nodes with identical metadata. Conversely, if ε*_ij_* = 1 for all *i* and *j*, then all transitions are encoded irrespective of the metadata.

To derive a closed-form expression of the metadata-dependent encoding graph, we start from the original graph *G* with link weights *w_kj_* between nodes *k* and *j*. Following the standard approach for discrete time random walks on weighted networks (*24*), we write the one-step transition probability that a random walker visiting node *k* will visit node *j* at the next step as

$$\pi_{\mathrm{jk}}=\frac{w_{\mathrm{kj}}}{\sum_{j}w_{\mathrm{kj}}}$$ (1)

We define the transition matrix to be column stochastic, because it simplifies the notation later. For the walks that restart after each encoding, we denote by *p_j_*(*t*∣*i*) the probability density of random walks restarting at node *i* at time 0 and being at node *j* at time *t*, so that *p_i_*(0∣*i*) = *p_i_* and

$$p_{j}(t|i)=\sum_{k}p_{k}(t-1|i)\pi_{\mathrm{jk}}(1-\varepsilon_{\mathrm{ik}})$$ (2)

accounts for all the possible ways in which a walker can jump to node *j* at time *t*, given that it did not encode on any of the neighbors of *j* at time *t* − 1. We can express the metadata-dependent encoding graph’s probability flows *e_ij_* as the time integral of the probability for a walker to encode at node *j* at any time *t* > 0 when starting from node *i* at time 0

$$e_{\mathrm{ij}}=\sum_{t=1}^{\infty }\varepsilon_{\mathrm{ij}}p_{j}(t|i)$$ (3)

With ${\tilde{\pi }}_{\mathrm{jk}|i}=\pi_{\mathrm{jk}}(1-\varepsilon_{\mathrm{ik}})$ for the probability to jump from node *k* to node *j* without encoding at *k* and ${\tilde{\Pi }}_{i}=\{{\tilde{\pi }}_{\mathrm{jk}|i}\}$, we can rewrite the master equation in Eq. 2 as

$$P(t|i)={\tilde{\Pi }}_{i}P(t-1|i)$$ (4)

where *P*(*t*∣*i*) is the column vector of node visit probabilities at time *t* when the walk started from node *i* at time *t* = 0. Equation 4 is formally identical to the master equation of a walker governed by the transition matrix $\tilde{\Pi }$, whose solution is

$$P(t|i)={\tilde{\Pi }}_{i}^{t}P(0|i)$$ (5)

with *P*(0∣*i*) = {δ*_ij_p_i_*}, where *p_i_* is the stationary occupation probability of the standard random walk without metadata-dependent encoding.

This result means that the transition weights of the metadata-dependent encoding graph in Eq. 3 can be rewritten as

$$E_{i}=\sum_{t=1}^{\infty }E_{i}^{⊤}{\tilde{\Pi }}_{i}^{t}P(0|i)$$ (6)

where E*_i_* ∀*i* ∈ *V* is the column vector of encoding probabilities for walkers starting at node *i*, and *E_i_* is the column vector of the probability flows of the metadata-dependent encoding graph *E* originating from node *i*. The metadata-dependent encoding graph *E* is a flow graph that represents the probabilities for a random walk to encode a transition between each node *i* and any of the *N* nodes of *G* such that ∑_*i*,*j*_*e_ij_* = 1 if the underlying graph *G* is connected. *E* is typically dense since *e_ij_* may be nonzero even if node *i* and node *j* are not directly connected by an edge.

The encoding graph *E* integrates structural and metadata information. Its properties depend on the structure of the underlying graph *G*, the distribution of metadata across the nodes, and the function used to determine the encoding probabilities {ε*_ij_*}. Given a graph *G* and node metadata, the encoding probabilities {ε*_ij_*} are the only free variables. Choosing those encoding probabilities, contingent upon the problem and question at hand, enables a continuous dependence of *E* on *G* and node metadata with categorical or scalar variables.

### Mapping random walks with metadata-dependent encoding probabilities

Any community detection algorithm that can handle graphs with directed, weighted links would, in principle, be able to identify meaningful dense subgraphs in the metadata-dependent encoding graph *E*. To identify communities of nodes that appear in relatively long uninterrupted encoding sequences of the random walk with metadata-dependent encoding probabilities, we use the map equation’s optimization method Infomap (*25*). To maximize the modular compression by minimizing the average description length given by the map equation, Infomap first searches for assignments of nodes into modules and then recursively searches for multilevel solutions. Since the metadata-dependent encoding graph comprises probability flows, we force Infomap to operate on the probability flows of *E* directly, without modeling flows on the input network, as the algorithm would normally do by default.

The random walk dynamics with metadata-dependent encoding probabilities generalizes the standard diffusion dynamics used by the map equation (*26*). Given a module partition ℳ of nodes in *G*, the map equation estimates the per-step average code length needed to describe movements of a random walk across the graph. In the original formulation, the map equation uses an index codebook to describe movements between communities and modular codebooks to describe movements inside communities. In this way, the map equation records transitions among nodes within the same community by using their module code words and transitions between nodes in different communities with three code words: one to exit from the current module codebook, one to enter the next module from the index codebook, and one for the destination node from the next module codebook. The standard map equation records every transition and depends only on the modular partition M and the flow graph defined by a random walk on *G*. Minimizing the description length of the movement of a random walker on a network over all possible partitions reveals the essential modular structure in the network for this metadata-ignorant walk.

When Infomap instead operates on the metadata-dependent encoding graph and exploits its dense subgraphs, the map equation effectively measures the per-encoding average code length of the random walk with metadata-dependent encoding probability on the original graph *G*. The communities that give the shortest code length reveal the essential modular structure of *G* and its distribution of metadata for this metadata-dependent walk, contingent on the choice of the encoding probability distribution {ε*_ij_*}.

### Encoding probabilities for categorical metadata

The simplest example that we consider is when each node is associated to a binary categorical variable such as high/low, rich/poor, ∘/•, and so on, and we assume that the probability ε*_ij_* for a walker to encode at *j* when starting from *i* depends only on the categories *f_i_* and *f_j_* to which *i* and *j* belong, respectively. Without loss of generality, we assume that the available categories are just {0,1} and set to

$$\varepsilon_{\mathrm{ij}}=p\delta_{f_{i},f_{j}}+\frac{p}{c}(1-\delta_{f_{i},f_{j}})$$ (7)

where *p* ∈ [0,1] and *c* ∈ [*p*, + ∞ ]. This assignment allows us to model assortative, neutral, and disassortative encoding probabilities

1) If *c* > 1, then the walker will encode more frequently at nodes belonging to the same class of the starting node (assortative encoding).

2) For *p* < *c* < 1, encoding will be more probable at nodes belonging to a different class than the one of the starting node (disassortative encoding).

3) For *c* = 1, the encoding dynamics no longer depend on class assignments (neutral encoding).

Irrespective of the value of *c*, the presence of a baseline encoding probability *p*, which, in general, is smaller than 1, means that the walker can traverse a large portion of the graph before terminating. In particular, in the limit *p* ≪ 1, the actual structure of the graph becomes less and less relevant, and the probability of a walker encoding at a given node is driven almost exclusively by categorical information. Conversely, when *p* ≃ 1, the probability of a walker encoding at *j* depends more on its distance from *i* on *G* than on metadata. In the special case where *p* = 1 and *c* = 1, the time scale of diffusion and encoding is the same, categorical information becomes irrelevant, and the encoding graph *E* coincides with the flow graph associated with a standard random walk without metadata-dependent encoding on the graph *G*, recovering the same behavior as the classical map equation.

**Fig. 3.**
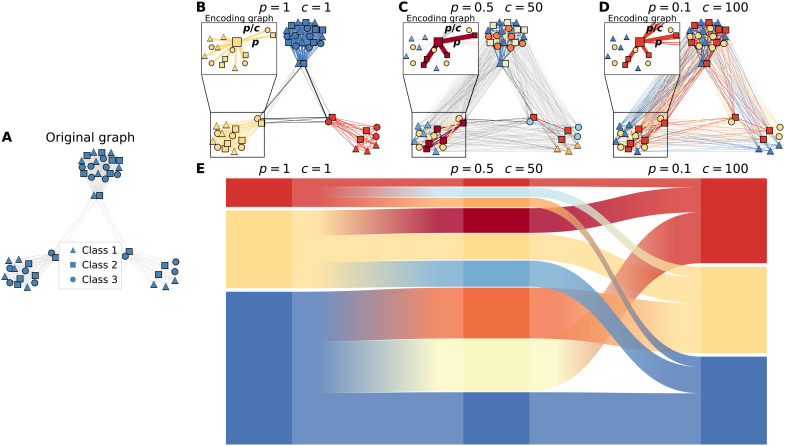
Metadata-informed graph communities in a synthetic network with cliques. (**A**) Original synthetic graph with three cliques $K_{9}$, $K_{15}$, and $K_{21}$ connected by a few intermodule links. Nodes are associated with three categorical metadata classes, indicated by squares, triangles, and circles. In each clique, one-third of the nodes belong to each of the three classes. Each panel shows the links in the encoding graph corresponding to a different pair of values *p* and *c*, where the inset details the probability of a walker to encode at a node of the same class (*p*) or of a different class (*p*/*c*) of the node where it started. (**B**) Community partitions obtained in the synthetic graph for *p* = 1 and *c* = 1, which correspond to the fully connected components that conform the network. For such values, all transitions are encoded, leading to a community partition equivalent to the original graph. (**C**) Community partitions obtained for *p* = 0.5 and *c* = 50, leading to a further division of the fully connected cliques into three more communities determined by node classes. (**D**) Community partitions obtained for *p* = 0.1 and *c* = 100. With a lower value of *p*, nodes of the same class belonging to different cliques are assigned to the same community, yielding a partition consisting of only three communities determined solely by metadata. (**E**) Alluvial diagram depicting changes in the detected communities. In (B) to (D), the links of the encoding graph have the same color as the nodes that they connect, if they belong to the same community, or are gray otherwise. The insets depict the links of the encoding graph for an example node, with linewidth proportional to the link weight.

The simple synthetic graph consisting of three loosely interconnected cliques in Fig. 3 shows an example of this assignment. We use encoding probabilities based on Eq. 7 also for categorical metadata with more than two categories. We consider real-valued metadata when the distance between two categories can be quantified in a meaningful way and includes potentially interesting relationships between metadata and network structure.

### Encoding probabilities for real-valued metadata

The second example that we consider is real-valued node metadata, where each node *i* is assigned a real number *f_i_* ∈ ℝ. We assume that the encoding probability ε*_ij_* is inversely proportional to ∣*f_i_* − *f_j_*∣, so that ε*_ij_* will be higher if *i* and *j* are associated with similar metadata values. In general, we could choose to modulate the encoding probability through any decreasing function of ∣*f_i_* − *f_j_*∣, but here, we consider only the probability

$$\varepsilon_{\mathrm{ij}}=s\;\text{exp}\;(\frac{-|f_{i}-f_{j}|}{b})p+(1-s)$$ (8)

where *p* is the probability to encode at *j* when *f_j_* = *f_i_*, *b* ∈ *R*^+^ is a scale parameter so that larger values of *b* correspond to higher encoding probability, and *s* ∈ [0,1] is the relative strength of metadata information. The complement of the metadata strength *s* is the baseline coding probability

s′=1−s (9)

where the probability to code when the distance ∣*f_i_* − *f_j_*∣ is large.

For *s* = 1, when the relative strength of metadata is maximal, Eq. 8 is a proper generalization of Eq. 7 for binary categories. In this case, we recover ε*_ij_* = *p* when *f_i_* = *f_j_* and ε*_ij_* = *p*/*c* when *f_i_* ≠ *f_j_*, with $c=e^{\frac{1}{b}}$. Equivalently, for standardized real-valued metadata with σ = 1, metadata values that are *b* SDs apart correspond to a binary separation of categories.

## RESULTS

We analyzed the range of community partitions found by the map equation’s search algorithm Infomap (*25*) on the encoding graphs of various synthetic and real-world systems. For each network, we constructed the encoding graph of Eq. 7 for different values of *p* and *c*. To illustrate how the encoding graph can integrate metadata and structural information, we start with a simple example: an unweighted synthetic network with three different classes equally distributed in three fully connected subgraphs with only a few links between them (Fig. 3A). In Fig. 3 (B to D), we show the partitions in communities identified by Infomap on the encoding graphs obtained for different values of *p* and *c*, corresponding to different ways of mixing structural and metadata information. When *p* = 1 and *c* = 1 (Fig. 3B), metadata play no role, and the best partition consists of three communities corresponding to the three cliques. As expected, this partition is identical to the one obtained by running Infomap on the original graph, and as for *p* = 1 and *c* = 1, the encoding graph is identical to the flow graph associated with a standard random walk without metadata-dependent encoding on the original graph *G*. Varying *p* and *c* strikes a balance between metadata and structure, obtaining other meaningful partitions. When *p* = 0.5 and *c* = 50, our method reveals a total of nine communities instead of just three communities (Fig. 3C). Each clique has been split into three submodules, corresponding to the nodes’ metadata assignments. In general, for large values of *p*, walkers encode before having the chance to move between communities. However, for sufficiently small values of *p*, walkers can move to other cliques and “see” other nodes belonging to the same class as that of their starting node. This leads to another meaningful partition where nodes are grouped by metadata values (Fig. 3D).

### Synthetic networks with planted community structure

In most real-world systems, available categorical information only partially correlates with network structure. Hence, it is important to assess the impact of strong and weak local correlations between metadata and network structure on the ability of the proposed method to detect meaningful metadata-informed partitions. Unlike traditional community detection algorithms, we do not seek to retrieve a ground truth, if such a thing exists, but to allow a wide range of node communities that depend on both the topology of the graph and the category of nodes. Ideally, the range of such partitioning should include the extreme cases where only topological features are considered or all nodes within the same category are grouped together. We expect that our framework is able to explore the full spectrum between these two extremes, taking into account the existing correlations between network structure and metadata and thus allowing somehow to quantify them. We start by considering a model to generate synthetic networks with *N* nodes, *N*_c_ communities and metadata categories, and *N*/*N_c_* nodes per community and category. We connect the communities in a ring-like shape so that a node *i* only connects to nodes belonging to its own community and to the two other communities adjacent to it. We tune the strength of the planted community partition by varying the probability *p*_1_ of creating a link between nodes in the same community and the probability *p*_2_ of creating a link between nodes in two adjacent communities. As *p*_2_ decreases relative to *p*_1_, the planted community structure becomes more pronounced.

**Fig. 4.**
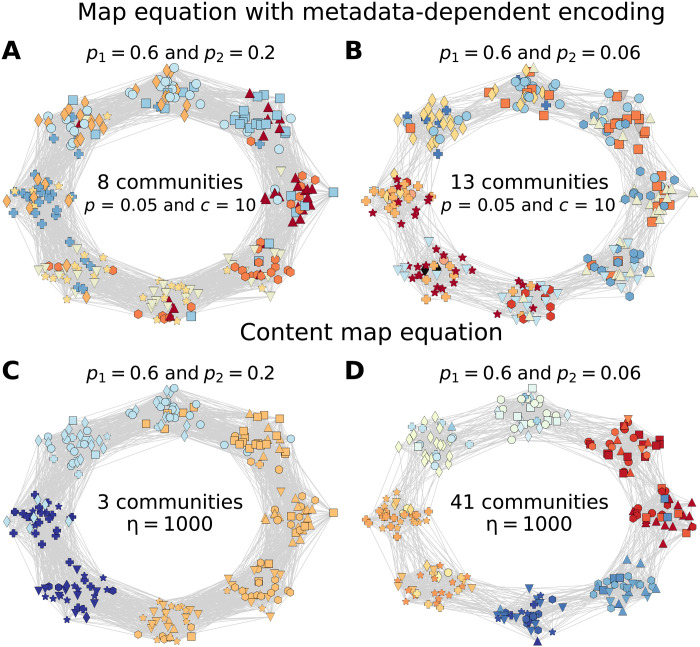
Metadata-informed graph communities obtained with the map equation with metadata-dependent encoding and the content map equation. (**A** and **B**) Partitions according to the map equation with metadata-dependent encoding for a network of 240 nodes with probabilities (A) *p*_1_ = 0.6 and *p*_2_ = 0.2 and (B) *p*_1_ = 0.6 and *p*_2_ = 0.06. (**C** and **D**) Partitions according to the content map equation for a network with probabilities (C) *p*_1_ = 0.6 and *p*_2_ = 0.2 and (D) *p*_1_ = 0.6 and *p*_2_ = 0.06. The color of the nodes corresponds to their community assignment, and the shape corresponds to their metadata category.

To provide tunable local correlations between network structure and metadata information, we start from a configuration in which the community structure matches the class distribution. All nodes of the same category belong to the same structural community. To explore the full range of possibilities between a perfect match of categories and communities and random class assignments, we progressively reshuffle the nodes’ associated metadata. Last, to allow for different degrees of nonlocal relationships between metadata and structure, we constrain the reshuffling to nodes with a community distance equal to *d*_c_. That is, only nodes that belong to communities separated by a distance *d*_c_ in the ring-like structure can reshuffle their metadata information (see Fig. 4).

To compare the results to those obtained by the content map equation (*18*), we start by setting *d_c_* = 1, meaning that metadata reshuffling only occurs across neighboring communities, and focus on two networks with probabilities (*p*_1_ = 0.6 and *p*_2_ = 0.2) and (*p*_1_ = 0.6 and *p*_2_ = 0.06). In Fig. 4, we show the partitions found by the two methods on the two synthetic graphs after 112 metadata reshuffling steps. Although *d*_c_ = 1 suggests that we focus on local correlations, iterated metadata reshuffling spreads categories across several communities. By looking at Fig. 4 (A and C), it is evident that the map equation with metadata encoding allows for a further grouping of nodes within the same category, while in most cases, the content map equation fails to group together nodes belonging to different structural communities. The recovery of the extreme regime not only is interesting in itself but also highlights how our approach recovers a whole range of partitions between the topological and the categorical communities. In particular, we observe in Fig. 4B how nodes of the same category are split into different communities if they appear to be far apart in the network, such as in the case of diamonds or crosses.

**Fig. 5.**
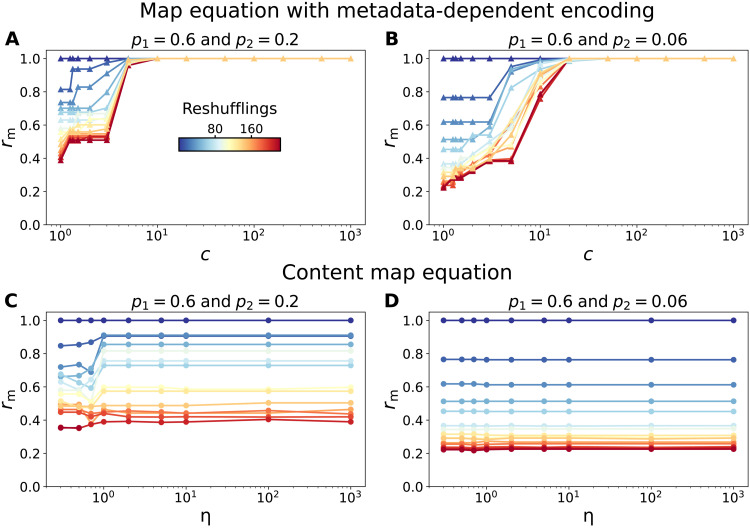
Evolution of the mixing ratio *r*_m_ for the map equation with metadata-dependent encoding and the content map equation as a function of the network structure and the correlation with metadata. (**A** and **B**) Mixing ratio *r*_m_ in the map equation with metadata-dependent encoding for *p* = 0.5 and increasing values of the metadata importance *c* in a network generated with (A) *p*_1_ = 0.6 and *p*_2_ = 0.2 and (B) *p*_1_ = 0.6 and *p*_2_ = 0.06. The colors indicate the number of metadata reshufflings going from low (blue) to high (red). (**C** and **D**) Mixing ratio *r*_m_ in the content map equation for increasing values of the metadata importance η in a network generated with (C) *p*_1_ = 0.6 and *p*_2_ = 0.2 and (D) *p*_1_ = 0.6 and *p*_2_ = 0.06.

To quantify the difference between the two algorithms, we define a mixing ratio based on the number of unique pairs of nodes of the same category that are placed in the same community by either algorithm (*27*, *28*)

$$r_{m}=\frac{1}{N_{c}}\sum_{k=1}^{N_{c}}\frac{a^{k}}{n_{k}(n_{k}-1)/2}$$ (10)

where *a^k^* is the number of pairs of nodes of category *k* that belong to the same community and *n_k_* is the total number of nodes of category *k*. The mixing ratio *r*_m_ = 1 when all the nodes of any category α are put into the same community, without exceptions. In Fig. 5 (A and B), we report the change of *r*_m_ with *c* when *p* = 0.5 for the map equation with metadata-dependent encoding in two networks generated with (*p*_1_ = 0.6 and *p*_2_ = 0.2) and (*p*_1_ = 0.6 and *p*_2_ = 0.2), respectively. The color gradient indicates the progressive metadata reshuffling, going from low (blue) to high (red). Our framework is not only capable of attaining the extreme case *r*_m_ = 1—all nodes of the same class in the same community—but also provides a wide variety of partitions in between. In fig. S1, we report the concrete partitions for several values of *c*, showing how nodes of the same category are first merged together in smaller close-by communities that are later unified as *c* increases. We can capture the interplay of network structure and metadata even when metadata association is not strongly linked with planted community partitions. Mixing nodes with equal metadata in different communities is achieved at lower values of *c* when *p*_2_ = 0.2 than when *p*_2_ = 0.06. Similarly, we also capture the stronger separation between categories obtained for a very large number of metadata reshufflings. Results for other values of *p* can be found in fig. S1. The content map equation, on the other hand, fails to join together nodes in separated communities as the connection between communities weakens (see Fig. 5, C and D) and can only merge them when the community structure is weak. If communities are loosely connected, then the content map equation provides little additional information, since it keeps splitting nodes of the same category in the same structural community.

We have also studied the effect of the distance at which reshuffling is allowed, *d_c_*, which allows us to tune the level of nonlocal relations between categories and planted communities. As we show in fig. S2, our approach is sensitive to the values of *d_c_*, as it requires lower values of *p* and higher values of *c* to merge nodes of a similar category when *d_c_* increases. The intermediate regimes also gradually vanish as *d_c_* increases, showing a good sensitivity to nonlocal relations. If nodes of different categories are located in two far-apart modules, then intermediate regimes are generally harder to find. We have also explored a simpler model in which the network is generated by walkers that either move through the graph or are teleported to nodes of the same metadata category. Despite such a framework being able to reproduce the extreme cases, it fails to take into account the cases in-between since the teleportation does not properly take into account the distance between nodes in the graph (figs. S3 and S4).

### Social contact networks

Several real-world contact networks have metadata attached to nodes, providing explicit information about the function or position of any given individual in the system. For instance, the metadata can identify the role of each node of a hospital contact network or the class to which students of a school belong. Taking this information into account can be crucial to interpreting how a system functions.

**Fig. 6.**
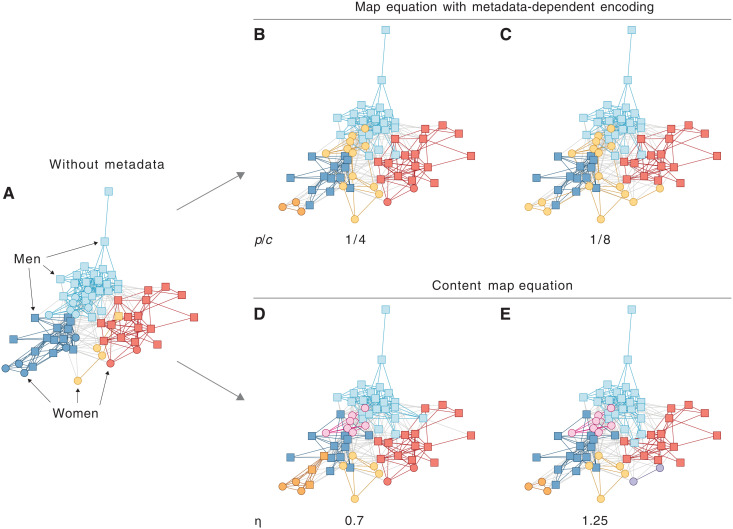
The optimal partitions of the Lazega lawyer’s friendship network. (**A**) With standard Infomap without gender information, the modules are solely determined by direct network links. (**B**) With random walks with encoding ratio *p*/*c* = 1/4, two women-only modules appear, and all women are included in three of the five modules. (**C**) With encoding ratio *p*/*c* = 1/8, all women appear in a separate module. The content map equation (*18*) gives similar modular structure for metadata rate η = 0.8 (**D**), which is further subdivided for metadata rate η = 1.25 (**E**).

**Fig. 7.**
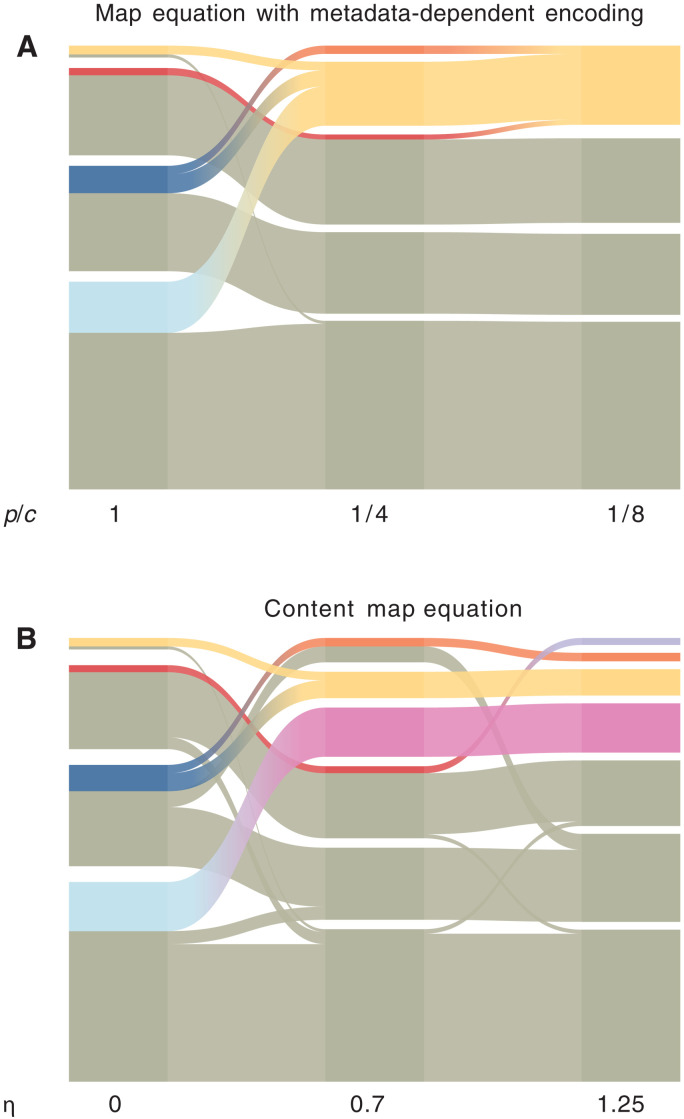
Alluvial diagrams of the Lazega lawyer’s friendship network. (**A**) Random walks with encoding ratios 1, 1/4, and 1/8 matching panels in Fig. 6 (B and C). (**B**) Using the content map equation with metadata rate η = 0, 0.7, and 1.25 matching panels in Fig. 6 (D and E). Encoding ratio 1 in (A) and metadata rate 0 in (B) discards the gender information and yields the same partition. The fraction of women in each module is colored according to the module assignments in Fig. 6.

As a first example, we considered the Lazega lawyers’ friendship network (*29*) with gender information. We report in Fig. 6 the partitions obtained by using different values of the parameters *p* and *c*. In Fig. 6A, *p* = 1 and *c* = 1, so that no gender information is considered and the partition relies purely on the structure of the graph. In Fig. 6 (B and C), the gender of each node increases in relevancy, leading to an almost complete separation between nodes of different genders in Fig. 6C. The transition between structure-focused and metadata-focused partitions is illustrated in the alluvial diagram in Fig. 7A. The map equation with metadata-dependent encoding puts female nodes in a community of their own for sufficiently low values of *p*/*c*, irrespective of the original structural community that they belonged to, thus pointing out that women might be more tightly connected compared to men. To compare our results with previous work, we reproduced the results obtained on the same graph using the content map equation, with metadata rates η = 0.7 and 1.25, respectively [see Fig. 3 and (*18*)]. In Fig. 7D, the women start to separate from the men but in different ways. Overall, increasing the metadata rate separates men and women but tends not to join back communities of the same gender. When the metadata rate η = 1.25 (Fig. 7E), men and women are completely separated, but same-gendered modules are never joined again. As before, the alluvial diagram in Fig. 7B shows how small submodules of female nodes are isolated from larger modules of male ones.

**Fig. 8.**
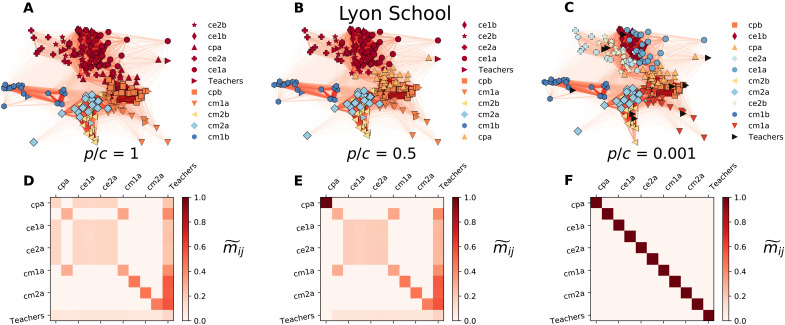
Metadata-based communities in the Lyon School contact network. Communities in the Lyon School contact graph, where nodes correspond to individuals and each node is assigned a label corresponding to the class it belongs to. Teachers are put in a separate class. The probability to encode a transition is *p* if both nodes belong to the same class and *p*/*c* otherwise. We show the results or *p* = 1 where *c* = 1 (**A**), *c* = 2 (**B**), and *c* = 1000 (**C**). (**D** to **F**) Class overlapping assignment ${\tilde{m}}_{\mathrm{ij}}$ when *c* = 1 (D), *c* = 2 (E), and *c* = 1000 (F). Nodes are colored according to their community assignment, while markers indicate their metadata information.

As another example of metadata-enriched social networks, we consider the Lyon School contact graph (*30*), which is one of several annotated social networks made available by the SocioPatterns project (*31*). The graph reports the face-to-face interactions among students in a school in Lyon. For each node, we know whether the corresponding person is a student or a teacher and to which class she belongs. In total, the dataset consists of a dense network with *N* = 242 nodes, *K* = 26,594 links, and 11 node classes (10 classrooms plus teachers). In Fig. 8, we display the partitions obtained for *p* = 1 and *c* = 1 (Fig. 8A), *c* = 2 (Fig. 8B), and *c* = 1000 (Fig. 8C). By increasing *c*, the class of each node becomes more relevant, and the structural communities start to split. Teachers are the last metadata class to be recovered, because the majority of each teacher’s face-to-face contacts happens with pupils in their respective class. To assess the extent to which nodes with different categorical metadata information are assigned to the same community, we use the class overlap *m*_αβ_ between classes α and β defined as

$$m_{\alpha \beta }=\frac{1}{N_{\alpha }+N_{\beta }}\sum_{c\in C}(N_{c\alpha }+N_{c\beta })(1-\delta_{N_{c\alpha },0}\delta_{N_{c\beta },0})$$ (11)

where C are the communities reported by our algorithm, *N*_α_ is the total number of nodes of class α, and *N*_*c*α_ is the total number of nodes of class α in module *c*. This quantity is equal to 1 when no community exists where there is a node of class α and no node of class β and vice versa for any possible choice of α and β. Conversely, *m*_αβ_ is equal to 0 when no node of class α appears in a community together with a node of class β. For easy comparison between different classes, the final quantity that we have considered is the row-normalized counterpart given by

$${\tilde{m}}_{\alpha \beta }=\frac{m_{\alpha \beta }}{\sum_{\beta }m_{\alpha \beta }}$$ (12)

This measure assesses how nodes with different (or equal) metadata information are assigned to the same community when the parameters of the metadata-dependent encoding are tuned. As depicted in Fig. 8 (D to F), by tuning *p* and *c*, we can infer the relation between groups and network topology, with more isolated groups such as cm2a or more interconnected such as ce2a. Similarly, the bottom row in Fig. 8 (D and E) evidences that the teacher category is more closely connected to the rest of the classes. The nodes with equal category end up in the same community when *c* is sufficiently large and the probability for a walker to encode at any node is relatively small. For additional results using two other social networks, see figs. S5 and S6.

### Socioeconomic indicators and mobility in London

**Fig. 9.**
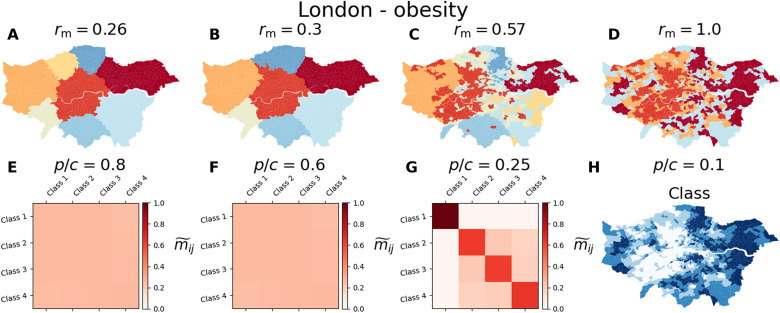
Partitions obtained for the obesity categories in the commuting network of London. Community detection analysis on the commuting network of London when the metadata are set according to the obesity category. Regions in classes 1 and 4 corresponding to those where obesity is less and more prevalent, respectively. For a probability *p* = 1, partitions when *c* = 1.25 (**A**), *c* = 1.66 (**B**), *c* = 4 (**C**), and *c* = 10 (**D**), with regions colored according to their community assignment. (**E** to **G**) Class overlapping ${\tilde{m}}_{\mathrm{ij}}$ when *c* = 1.25 (E), *c* = 1.66 (F), and *c* = 4 (G). (**H**) The class assignment for each of the regions studied.

In spatial systems, networks can be defined in multiple ways, not only according to the distance separating two regions but also according to the number of people who move between places. We show the partitions in communities obtained for the mobility network of London, where the relevant metadata of a region are set according to a variety of socioeconomic indicators. We considered Greater London at the level of Middle Layer Super Output Areas (MSOAs), where each MSOA is a node, and the weighted network connecting MSOAs is based on the mobility of commuters. The commuting data include the number of individuals T*_ij_* who live in a spatial unit (MSOA) *i* and work in a spatial unit *j*. From those commuting patterns, we built a mobility network between MSOAs in which the weight of a link going from a spatial unit *i* to a spatial unit *j* is given by *w_ij_* = T*_ij_* + T*_ji_*. The graph is then a reflection of the back-and-forth trips performed by working individuals. While the graph produced is directed, in practice, we can state that *w_ij_* = *w_ji_* by construction. We have performed a set of simulations in which the node categories are set to median income, obesity rate, life expectancy, overall health condition, socioeconomic deprivation, the fraction of white individuals, and unemployment. In each case study, nodes are associated with one of four classes depending on the quartile of the distribution to which they belong. A variety of patterns emerge depending on the correlation between mobility and the spatial patterns of each indicator. The results for obesity rates are reported in Fig. 9 for *p* = 1 and *p*/*c* = 0.8 (Fig. 9A), *p*/*c* = 0.6 (Fig. 9B), *p*/*c* = 0.25 (Fig. 9C), and *p*/*c* = 0.1 (Fig. 9D) together with the corresponding values of *r*_m_. Each class represents a quartile of the distribution, with class 1 corresponding to regions where obesity is less prevalent and class 4 where it is more prevalent. By increasing the relative importance of metadata (Fig. 9H), we obtain substantial changes in the partitions. In particular, when *c* is small, the modules are effectively determined mainly by spatial distance. As *c* increases so does *r*_m_, and areas of the same category start to merge together, either in large communities when they are close by or many smaller ones when they are far apart. For instance, the regions with low rates of obesity are much more concentrated in the eastern side of the city and thus are much more easily merged together. On the other hand, those regions where obesity is more prevalent are more scattered around and are first merged into separated modules. The changes in the partitions can be more clearly observed in Fig. 9 (E to G), where the increase of *c* affects the number of MSOAs of the same income category assigned to the same community. Last, for a sufficient large value of *c* (Fig. 9D), all the regions with the same category are merged in the same module. In figs. S8 to S12, we show similar results for other socioeconomic indicators.

We have analyzed other spatial systems such as the ethnic segregation in Detroit and the spatial distribution of venues in cities. Unlike the case of London, for Detroit, we considered the network of adjacent census tracts and assigned a category as a function of the most overrepresented ethnicity (fig. S13). Given the strong correlation between the network of spatially adjacent regions and their ethnicity—census tracts with the same ethnicity tend to be closer to each other—we observe a wide range of partitions between the two extreme regimes. Neighborhoods are first defined by spatial proximity and, as *c* increases progressively, divide depending on the cell categories. We have also analyzed the spatial distribution of the different types of venues in Barcelona, Prague, and Berlin (figs. S14 to S16) (*32*–*34*). Close-by venues are connected in a spatial graph, and node class is set according to the different types of venues. For low values of *c*, close-by venues are grouped together regardless of their type, but by increasing *c*, the modules begin to blur depending on the types of venues around and end up splitting. Our methodology is a tool to identify neighborhoods of same-type venues for a proper choice of *p* and *c*.

### Power grid network

**Fig. 10.**
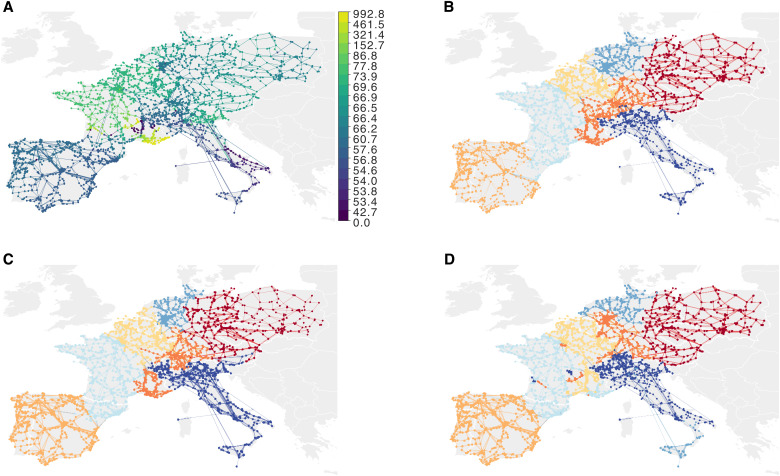
European power grid network with node prices and optimal partitions. (**A**) Node prices (€) distribute with lower prices in southern and central Europe and higher in western Europe. (**B**) With metadata strength *s* = 0, modules mix high- and low-price regions. In (**C**) and (**D**), node prices influence the partitions. (C) With *s* = 0.3, the module dominated by Italy grows to include the low-price region in eastern France and the module dominating eastern Europe grows to the west. (D) With *s* = 1, the red module containing northeastern France and Belgium grows to include the low-price region in southern France, dividing the higher-priced surrounding regions. Sicily splits from the lowest-priced regions of Italy.

As a final example, we consider the European electrical power grid, which forms a transport network connecting electricity producers and consumers. This system is structured similarly to a road transport network, with “highways” between large hubs connecting smaller neighboring cities. Electricity can flow instantly all across Europe, making it a large and dynamic system to consider as a whole. Each node has an associated electricity price. The price distribution is relatively heterogeneous in the analyzed situation, with low-price regions mainly around southern Europe where there is high solar production, average-price regions in central Europe, and high-price regions in western Europe (Fig. 10A). Historically, country borders defined energy market bidding zones, but to streamline these bidding zones over more coherent and connected price zones, European institutions are striving to revise them. As a result, prices correlate in space, but price ranges do not strictly map into countries or other political divisions.

To include real-valued prices as metadata in the random walk encoding, we derived the coding probability using the standardized price distance between nodes (Eq. 8) with scale parameter *b* = 0.5. To achieve ergodic visit rates, we started 10^7^ random walks per node for different metadata strengths *s* varying between 0 and 1.

We ran 100 optimization trials with Infomap for each metadata strength. The resulting partitions have six or seven levels of nested modules and organized into six or seven top-level super modules. Because we are interested in the grid’s large-scale organization, we restricted Infomap to search for partitions with seven top-level modules, again using 100 trials.

Increasing the metadata strength from *s* = 0 to *s* = 1 shifts the power grid communities from geographic to energy price–coherent modules. With metadata strength *s* = 0, the resulting modules map to densely connected regions such as Spain, Italy, western France, and eastern Europe. Germany and the Benelux countries divide into three modules, including the higher-priced region in southern France (Fig. 10B). When *s* = 0.3, the module containing Italy forms a wedge that divides the module with higher prices containing southern Germany and southern France, and the eastern Europe module grows into northern Germany (Fig. 10C). Last, with *s* = 1, several modules form distant colonies in similar-priced areas. The module dominated by southern Germany grows toward the Benelux countries with small colonies in the module dominated by central France, which, in turn, forms a colony in the higher-priced region in southern France. With its long-range connections, Sicily splits from the lowest-priced regions of Italy and joins northern Germany (Fig. 10D). Overall, incorporating metadata when identifying communities in the power grid network provided a more nuanced picture of the relations between energy price and geography beyond country borders.

## DISCUSSION

We have shown how to include metadata in the map equation using random walks with metadata-dependent encoding probabilities to integrate structural and metadata information beyond nodes’ immediate neighbors. By coupling the random walk encoding probability to the nodes’ metadata, we provide a tunable encoding scheme: The metadata modulate a walker’s coding horizon, simultaneously accounting for the node classes’ distribution and their relationships in the network. This approach equips researchers with a tool for identifying mesoscale structures in networks based on link structure and discrete or continuous node metadata information.

Metadata about the components of a system can be as relevant as knowing how those components connect. A researcher must use her application-specific knowledge when deciding how much metadata information she should include to identify functional modules of a complex system. The few community detection algorithms that consider metadata impose heavy constraints either on the relative role of structure and metadata or how they use metadata. For instance, current algorithms for detecting flow-based communities can only further divide structural communities into smaller metadata-based submodules.

To enable related submodules to merge or groups of nodes to move from one module to another based on their metadata, we propose a simple formalism that modifies the encoding procedure of the map equation. Metadata-dependent encoding probabilities at each node induce an encoding graph that effectively combines structural and metadata information about a network. The encoding graph is in itself an effective way to merge structural and metadata information about a network. While any community detection algorithm for weighted and directed links can operate on the encoding graph, using Infomap with its map equation objective function provides a simple interpretation: communities that compress a modular description of random walks with metadata-dependent encoding probabilities.

Tunable encoding probabilities allow the researcher to easily incorporate specific field knowledge in community detection. Our examples show how different values of *p* and *c* give various relevant solutions for categorical metadata. In general, larger values of *c* let walkers visit larger portions of the graphs before encoding, allowing relatively distant nodes with similar metadata to cluster together. Similarly, smaller values of *p* tend to yield larger communities. Researchers benefit from exploring the parameter space and selecting the most meaningful ranges of *p* and *c* that provide informative partitions for their particular problem.

Our compression-based approach opens a new avenue for community detection by linking network structure and metadata through the encoding of random walks on networks. Various information about nodes and edges, such as the physical location of nodes, edge classes, or other exogenous classification and rankings, provides enticing directions for new insights into complex systems.

## Supplementary Materials

### This PDF file includes:

Supplementary Note S1 Sections S1 to S6 Figs. S1 to S16
