# Supplementary material for: Mapping nonlocal relationships between metadata and network structure with metadata-dependent encoding of random walks
Source: Sci Adv. 2022 Oct 28;8(43):eabn7558. doi: 10.1126/sciadv.abn7558 (PMC9616498; doi:10.1126/sciadv.abn7558)
Supplement: Supplementary file 1 — Supplementary Note S1 Sections S1 to S6 Figs. S1 to S16 [file sciadv.abn7558_sm.pdf]

Science Advances
AAAS

Supplementary Materials for

Mapping nonlocal relationships between metadata and network structure with metadata-dependent encoding of random walks

Aleix Bassolas *et al.*

Corresponding author: Vincenzo Nicosia, v.nicosia@qmul.ac.uk

Sci. Adv. **8**, eabn7558 (2022)
DOI: 10.1126/sciadv.abn7558

This PDF file includes:

Supplementary Note S1
Sections S1 to S6
Figs. S1 to S16

Supplementary Note 1

In the current implementation of Infomap the parameter `--flow-model rawdir` skips the flow modeling step.

Section S1: Map equation with metadata-dependent encoding in synthetic graphs

In the main text we only show the partitions obtained for the Map Equation with metadata-dependent encoding in two cases. We provide in Fig. S1 further partitions in the intermediate regime in a network with $p_1 = 0.6$ and $p_2 = 0.06$ for $p = 0.5$. More concretely we show (**a**) c=1, (**b**) c=7, (**c**) c=8, (**d**) c=10 and (**e**) c=100. For completeness panel **f** has the corresponding alluvial plot.

For visualization purposes Fig. 4 of the main text only focuses on the $p = 0.5$ case, yet we provide in Figure S2 the results for a wider range of p and c. Given the higher complexity of our framework, we focus in only two cases per network: one with a low (**a,b**) and another with high (**c,d**) reshuffling. The first notable difference with Fig. 4 of the main manuscript, is that in all cases our framework can recover $r_m = 1$ with a smart choice of parameters. However, more interesting than the mere recovery on the extreme scenario, we can attain a far wider variety of partitions in-between. Our framework is also sensible to the network structure and the heterogeneity in the presence of metadata. Mixing together nodes with equal metadata in different communities is far easier (low values of c) when $p_2 = 0.2$ than when $p_2 = 0.06$. Similarly, we also capture the stronger separation between categories produced by more reshufflings.

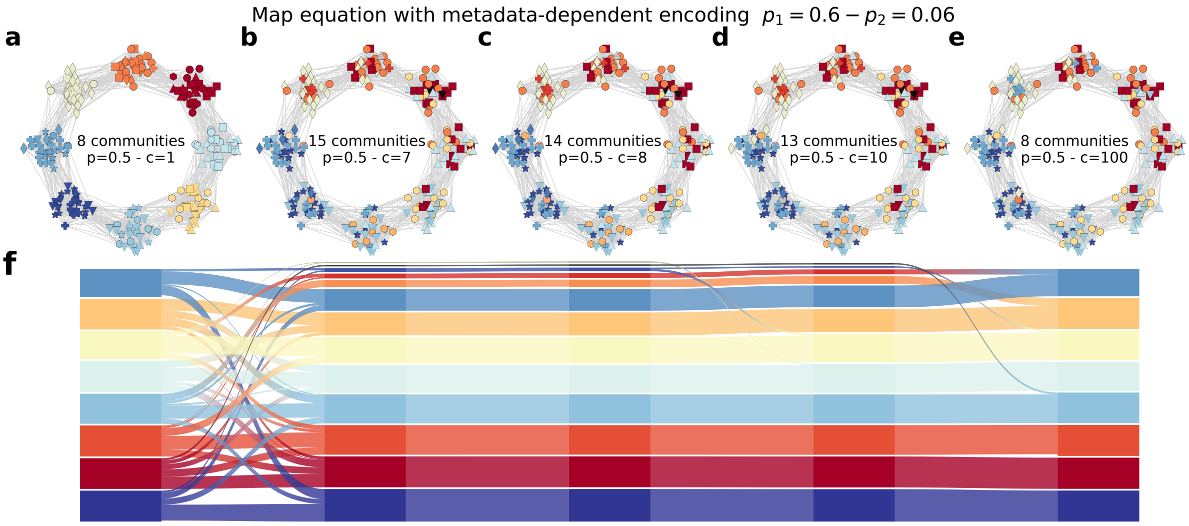

Figure S1: **Communities obtained with the map equation with metadata-dependent encoding in synthetic graphs. a-e** Partitions according to the map equation with metadata-dependent encoding for a network of 240 nodes with probabilities $p_1 = 0.6$ and $p_2 = 0.06$. In all cases $p = 0.5$ an **a** $c = 1$, **b** $c = 7$, **c** $c = 8$, **d** $c = 10$, and **e** $c = 100$. The color of the nodes correspond to their community assignment and the shape to their metadata category.

The reshuffling of metadata occurs hitherto between nodes in neighboring communities ($d_c = 1$), yet we could extend it to include larges distances and assess if our framework can detect non-local correlations in the presence of metadata. In Fig. S3, we display the values of r_m as function of p and c in four networks with an equivalent amount of randomizations ($n_r = 112$) but where the distance d_c is set to either 1, 2 3 or 4. As it can be seen, different distances yield different patterns of values in the (p,c) space. When d_c, it becomes more difficult to merge nodes of different categories into the same communities, or in other words, depending on the values raised by the combination of probabilities we can assess how are the metadata categories distributed.

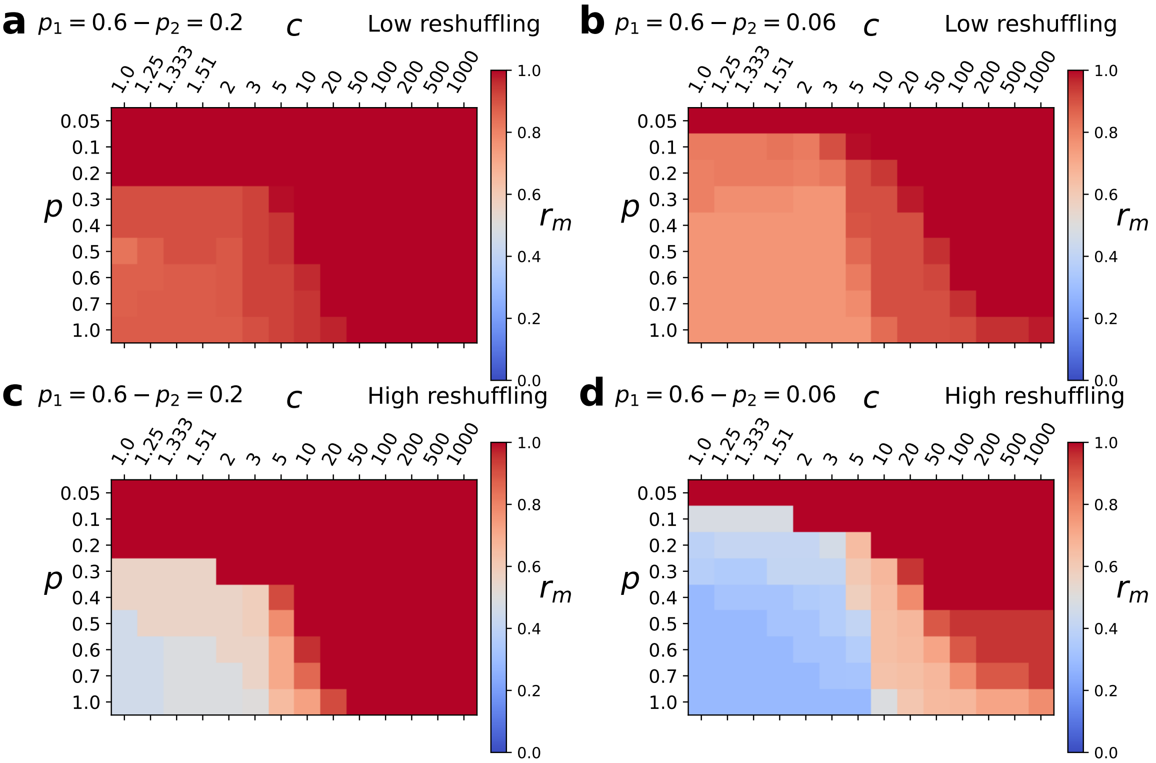

Figure S2: **Evolution of r_m in the map equation with metadata-dependent encoding depending on the network structure and the correlation with metadata. a,c** Mixing ratio r_m as a function of p and c for (**a**) a low and (**c**) high metadata reshuffling in a network generated with $p_1 = 0.6$ and $p_2 = 0.2$. **b,d** Mixing ratio r_m as a function of p and c for (**b**) a low and (**d**) high metadata reshuffling in a network generated with $p_1 = 0.6$ and $p_2 = 0.06$.

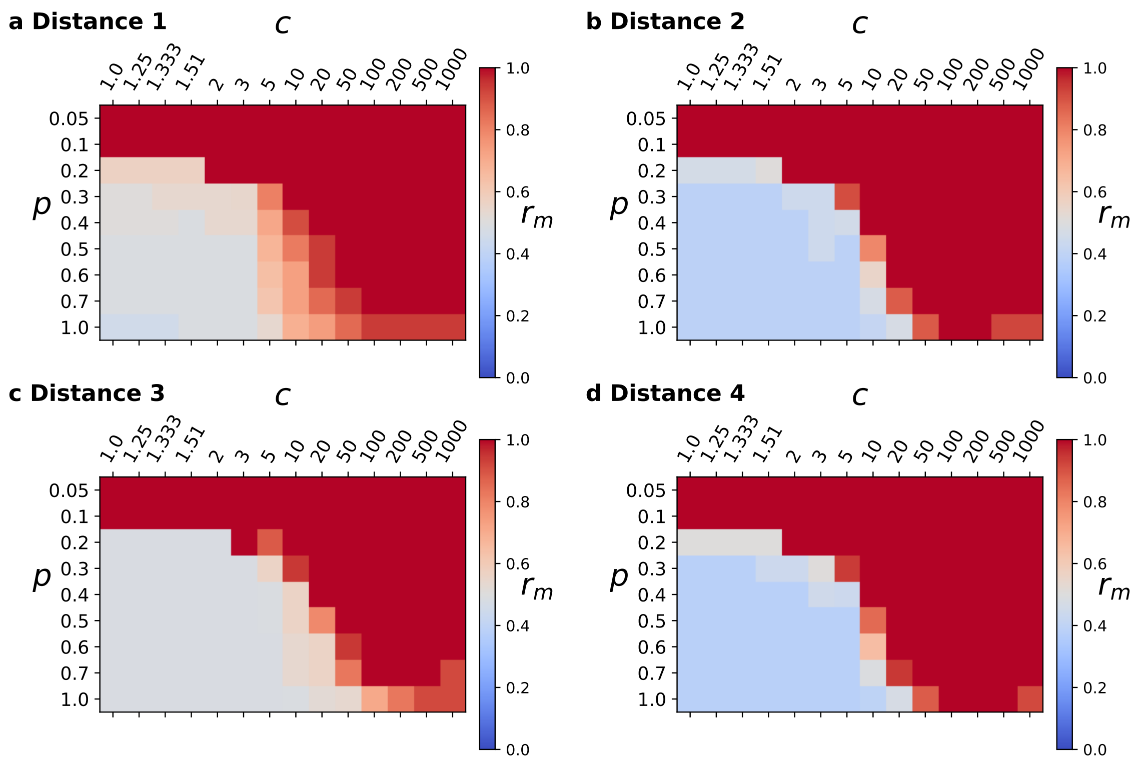

Figure S3: **Evolution of r_m in the map equation with metadata-dependent encoding depending on the correlation between structure and metadata. a-d** Mixing ratio r_m as a function of p and c for in a network generated with $p_1 = 0.6$ and $p_2 = 0.2$ when the metadata reshuffling occurs across communities separated by (**a**) $d_c = 1$, (**b**) $d_c = 2$, (**c**) $d_c = 3$ and (**d**) $d_c = 4$ in a ring-like topology.

Section S2: Map equation with class dependent teleportation

We investigate here if similar results can be recovered using more straightforward approach in which walkers move either through the real network or get teleported to another node with a class dependent probability. In detail, walkers have a probability p_n of moving through the network and a probability $1-p_n$ of being teleported, if it is the latter case, the walker have a probability p_c of moving to a node of the same category and $1-p_c$ of moving towards one of different category. In Fig. S4 we display the partitions obtained with four combinations of the probabilities p_n and p_c. When p_n is high (**a**), we retrieve the topological communities while as p_n decreases and p_c is high, the nodes with the same metadata information are assigned to the same community (**c**). Finally, in the extreme case in which p_n and p_c are low, all the nodes are assigned to the same community (**d**).

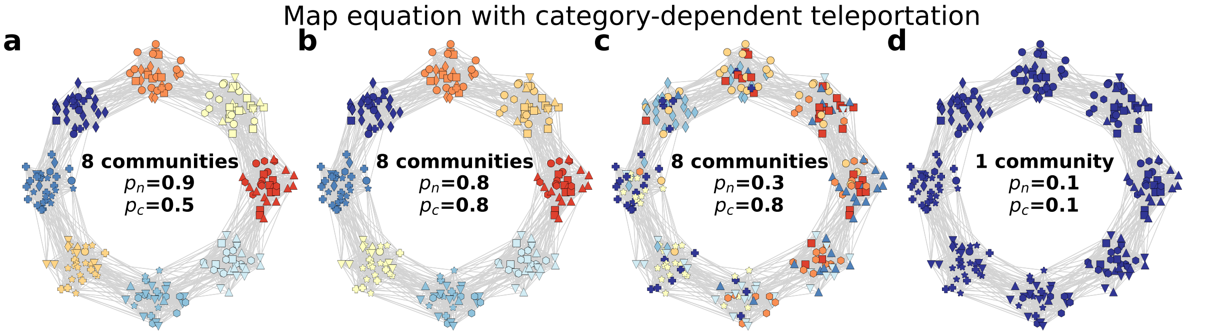

Figure S4: **Metadata informed graph communities obtained with the map equation with category dependent teleportation. a-d** Graph partition for a network with ($p_1 = 0.6$,$p_2 = 0.06$) according to the map equation with category teleportation with probabilities (**a**) ($p_n = 0.9$,$p_c = 0.5$), (**b**) ($p_n = 0.8$,$p_c = 0.8$), (**c**) ($p_n = 0.3$,$p_c = 0.8$) and (**d**) ($p_n = 0.1$,$p_c = 0.1$). All of them are calculated after $n_r = 112$ category randomizations

We further analyze in Fig. S5 how the mixing r_m changes as a function of the probabilities p_n and p_c for high and low reshufflings in a similar fashion to Figs. S2 and S3. Instead of the gradual classification we obtain with the map equation with metadata-dependent encoding, there is a dichotomous-like behavior that goes from either a split of nodes according to the structural communities or to their metadata information. When p_c is small we also observe that all the nodes fall within the same community. This behavior is likely caused by the fact that the teleporting only takes into account the metadata information regardless of the topological distance between nodes, with the exception of the first neighbors.

Section S3: Additional results for contact networks

We provide in Figs. S6 and S7 additional results of the communities detected for a workplace (InVS13) and a Hospital (LH10).

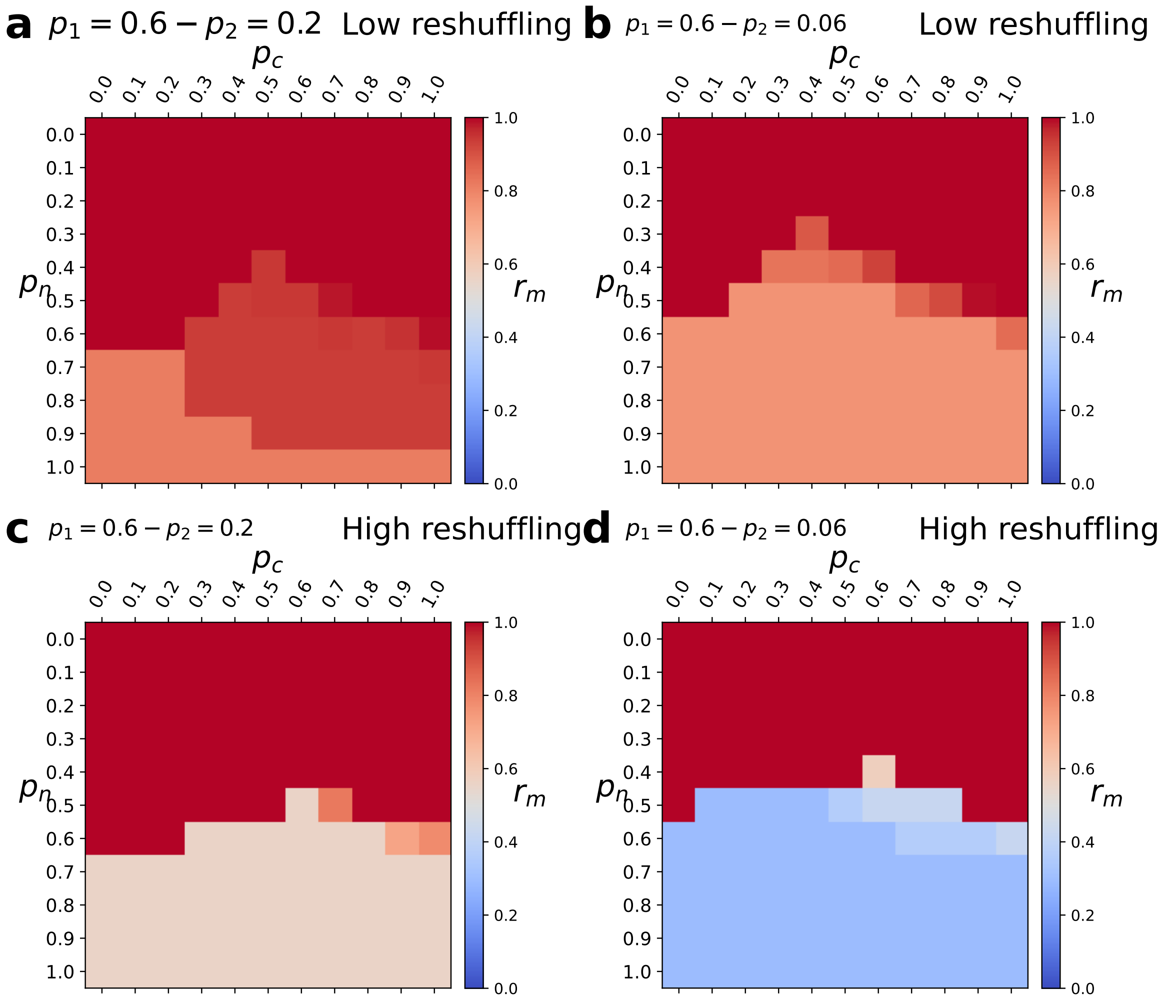

Figure S5: **Evolution of r_m in the map equation with category dependent teleportation. a,c** Mixing ratio r_m as a function of p_n and p_c for (**a**) a low and (**c**) high metadata reshuffling in a network generated with $p_1 = 0.6$ and $p_2 = 0.2$. **b,d** Mixing ratio r_m as a function of p and c for (**b**) a low and (**d**) high metadata reshuffling in a network generated with $p_1 = 0.6$ and $p_2 = 0.06$.

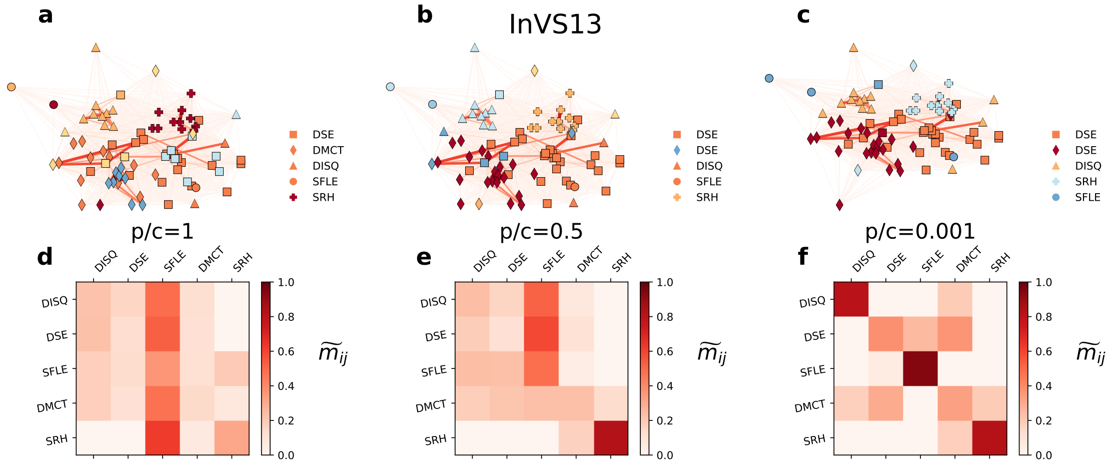

Figure S6: **Partitions obtained for the Workplace(InVS13) contact network.** Community detection analysis in the Lyon School contact graph where nodes correspond to individuals with a meta-data information. The probability to encode a transition is p for nodes within the same class and c otherwise. For a probability $p = 1$, partitions when $c = 1$ (**a**), $c = 2$ (**b**) and $c = 1000$ (**c**). **d-f** Class overlapping assignment $\widetilde{m}_{ij}$ when $c = 1$ (**d**), $c = 2$ (**e**) and $c = 1000$ (**f**). Nodes are colored according to their community assignment while markers indicate their meta-data information.

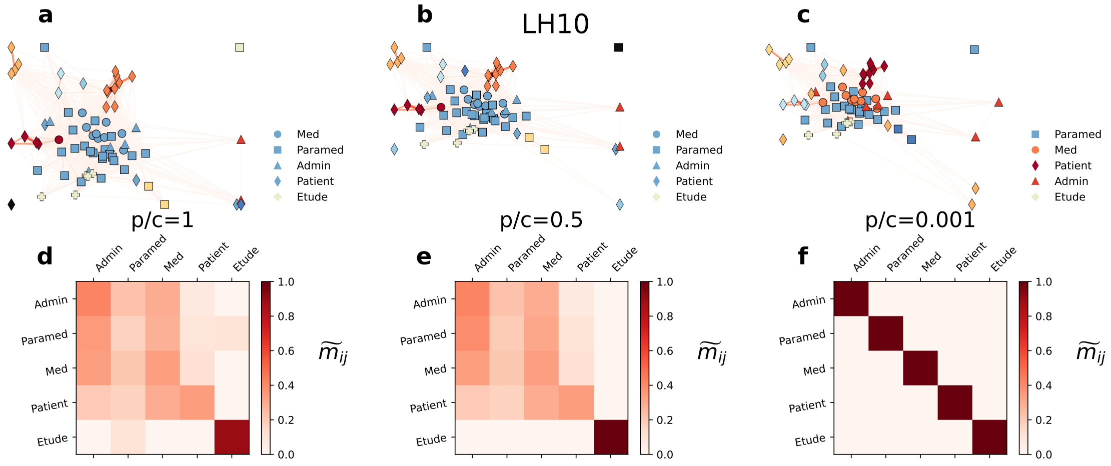

Figure S7: **Partitions obtained for the Hospital (LH10) contact network.** Community detection analysis in the Hospital (LH10) contact graph where nodes correspond to individuals with a meta-data information. The probability to encode a transition is p for nodes within the same class and c otherwise. For a probability $p = 1$, partitions when $c = 1$ (**a**), $c = 2$ (**b**) and $c = 1000$ (**c**). **d-f** Class overlapping assignment $\widetilde{m}_{ij}$ when $c = 1$ (**d**), $c = 2$ (**e**) and $c = 1000$ (**f**). Nodes are colored according to their community assignment while markers indicate their meta-data information.

Section S4: Additional results for the commuting network of London

We provide in Supplementary Figs. S8, S9, S11, S12 and S10 additional results for the London commuting graph when classes are assigned according to unemployment, life expectancy, deprivation, fraction of white individuals and obesity respectively.

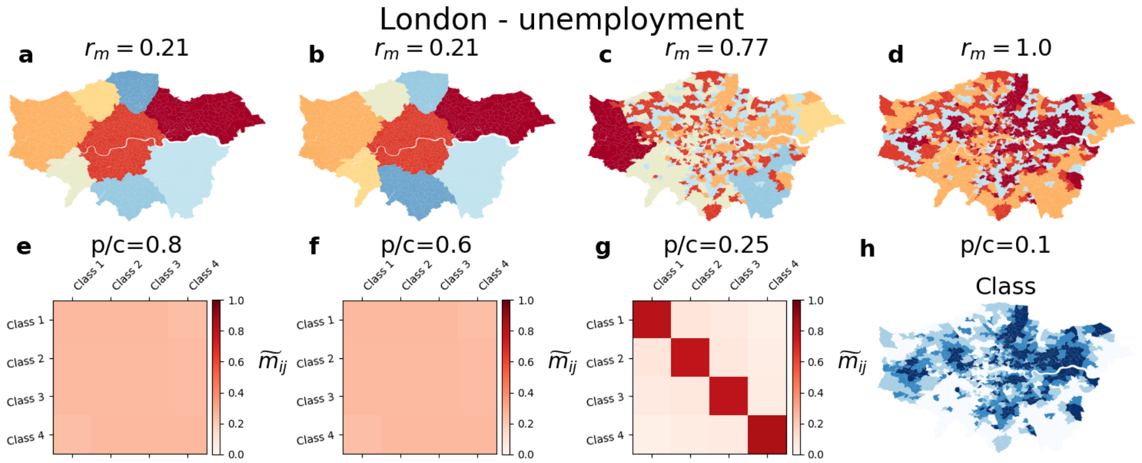

Figure S8: **Partitions obtained for the unemployment categories in the commuting network of London.** Community detection analysis on the commuting network of London when the metadata is set according to the unemployment category. With regions in class 1 and 4 corresponding to the last and most wealthy, respectively. For a probability $p = 1$, partitions when $c = 1$ (**a**), $c = 2$ (**b**) and $c = 1000$ (**c**), with regions colored according to their community assignment. (**d**) Class assignment for each of the regions studied. **e-g** Class overlapping $\widetilde{m}_{ij}$ when $c = 1$ (**e**), $c = 1.5$ (**f**) and $c = 1000$ (**g**).

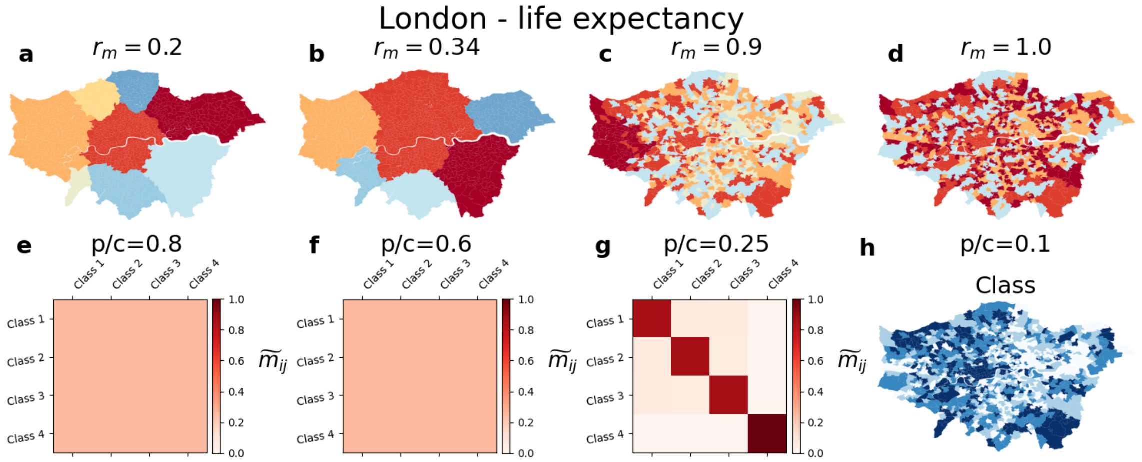

Figure S9: **Partitions obtained for the life expectancy categories in the commuting network of London.** Community detection analysis on the commuting network of London when the metadata is set according to the life expectancy category. With regions in class 1 and 4 corresponding to the last and most wealthy, respectively. For a probability $p = 1$, partitions when $c = 1.25$ (**a**), $c = 1.66$ (**b**) and $c = 4$ (**c**), with regions colored according to their community assignment. (**d**) Class assignment for each of the regions studied. **e-g** Class overlapping $\widetilde{m}_{ij}$ when $c = 1.25$ (**e**), $c = 1.66$ (**f**) and $c = 4$ (**g**).

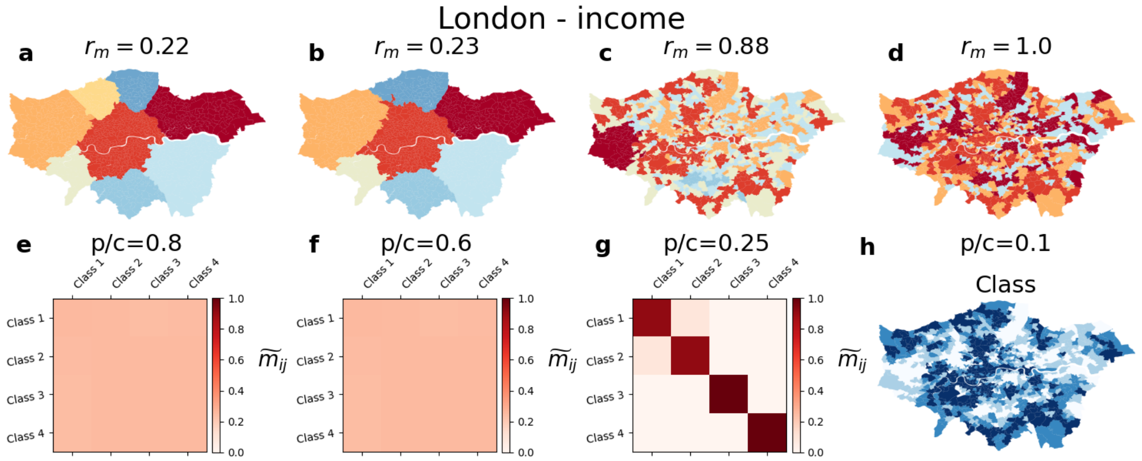

Figure S10: **Partitions obtained for the income categories in the commuting network of London.** Community detection analysis on the commuting network of London when the metadata is set according to the income category. With regions in class 1 and 4 corresponding to the last and most wealthy, respectively. For a probability $p = 1$, partitions when $c = 1.25$ (**a**), $c = 1.66$ (**b**) and $c = 4$ (**c**), with regions colored according to their community assignment. (**d**) Class assignment for each of the regions studied. **e-g** Class overlapping $\widetilde{m}_{ij}$ when $c = 1.25$ (**e**), $c = 1.66$ (**f**) and $c = 4$ (**g**).

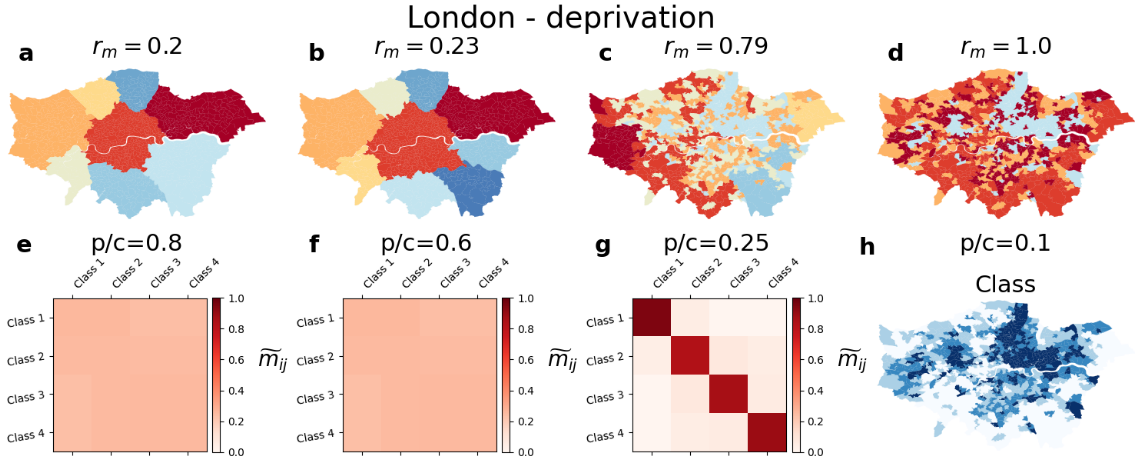

Figure S11: **Partitions obtained for the deprivation categories in the commuting network of London.** Community detection analysis on the commuting network of London when the metadata is set according to the deprivation category. With regions in class 1 and 4 corresponding to the last and most wealthy, respectively. For a probability $p = 1$, partitions when $c = 1.25$ (**a**), $c = 1.66$ (**b**) and $c = 4$ (**c**), with regions colored according to their community assignment. (**d**) Class assignment for each of the regions studied. **e-g** Class overlapping $\widetilde{m}_{ij}$ when $c = 1.25$ (**e**), $c = 1.66$ (**f**) and $c = 4$ (**g**).

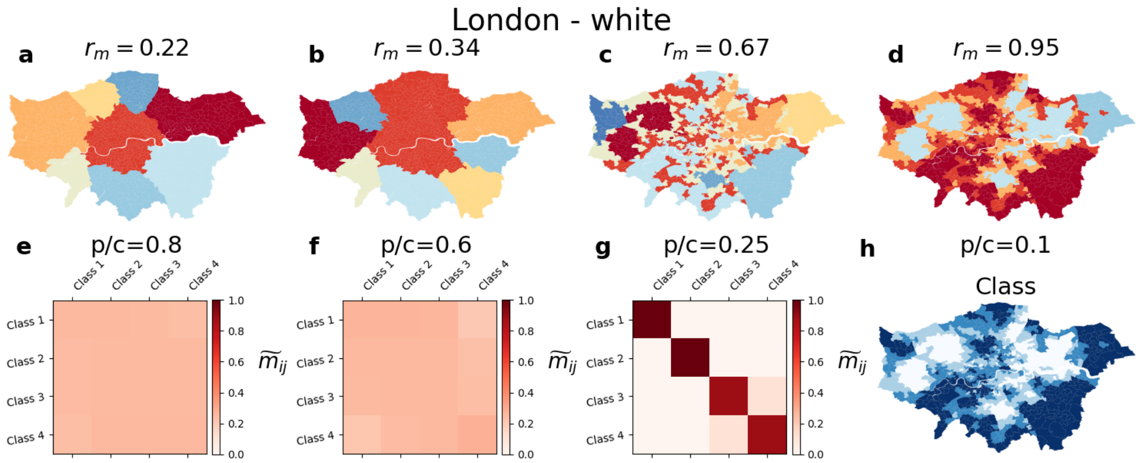

Figure S12: **Partitions obtained for the fraction of white individuals in the commuting network of London.** Community detection analysis on the commuting network of London when the metadata is set according to the fraction of white individuals category. With regions in class 1 and 4 corresponding to the last and most wealthy, respectively. For a probability $p = 1$, partitions when $c = 1.25$ (**a**), $c = 1.66$ (**b**) and $c = 4$ (**c**), with regions colored according to their community assignment. (**d**) Class assignment for each of the regions studied. **e-g** Class overlapping $\widetilde{m}_{ij}$ when $c = 1.25$ (**e**), $c = 1.66$ (**f**) and $c = 4$ (**g**).

Section S5: Ethnic segregation in Detroit

In this section we investigate the emergence of different neighborhoods in Detroit according to our metadata-dependent encoding scheme in the adjacency graph of spatial cells where categories obey to ethnicities. The network employed connects two cells, in this census tract units, when they are adjacent. The ethnicity with a higher relative abundance, calculated as the ratio between the number of residents of an ethnicity α divided by the city average, is assigned to each cell. The results for $p = 0.01$ and different values of c

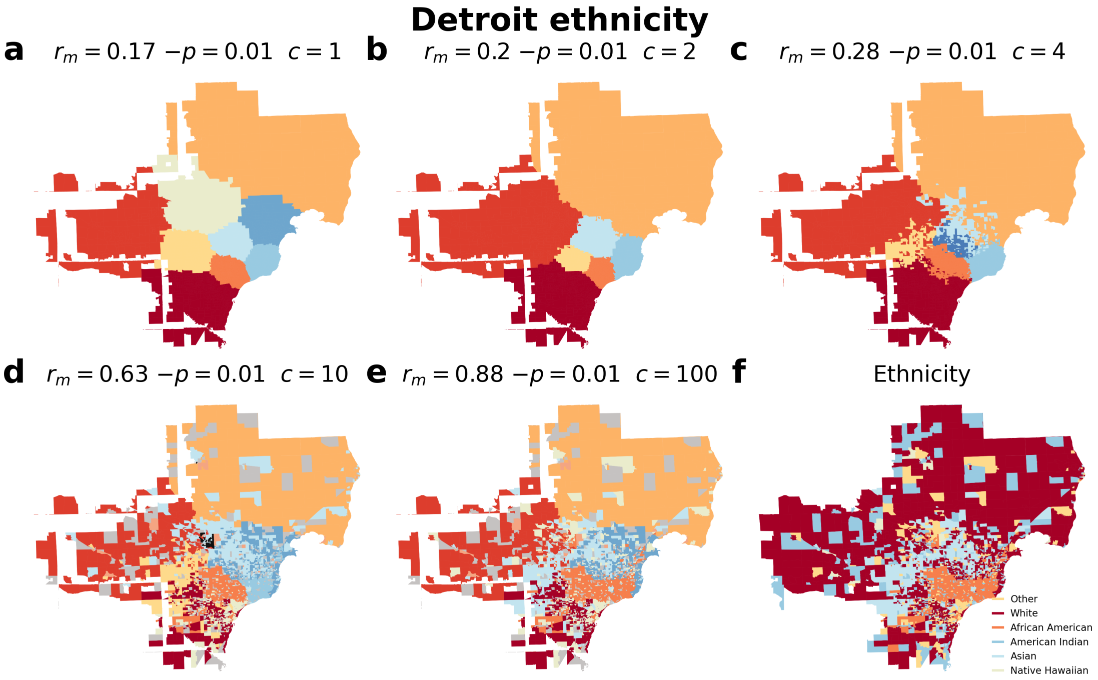

Figure S13: **Partitions obtained for the ethnicity categories in the spatial adjacency network of Detroit.** Community detection analysis on the spatial adjacency network of Detroit when the metadata is set according to the most overrepresented ethnicity. For a probability $p = 0.01$, partitions when $c = 1$ (**a**), $c = 2$ (**b**), $c = 4$ (**c**), $c = 10$ (**d**), $c = 100$ (**e**), together with the corresponding value of r_m. Panel **f** displays the category of each region.

Section S6: Organization of activities in urban areas

The proposed methodology can help identify functional modules in spatial systems. Standard community detection algorithms often do not provide the desired results on spatially-embedded networks. The spatial constraints are too strong to allow communities whose nodes are too far apart from each other. However, metadata is often available in spatial networks, and taking this information into account when detecting modules is desirable in many concrete applications. Typical examples include analyzing spatial correlation in the distribution of certain commercial activities or identifying spatial segregation according to a specific socio-economic indicator.

We consider a spatial data set constructed from the location-based social network Gowalla [33,34], which includes the location and type of millions of venues across the world. Whereas each venue has multiple classes organized hierarchically in this data set, we have only analyzed the main six categories: food, nightlife, outdoors, community, entertainment, and travel. The graph connecting the venues is spatial, there is a link between any pair of venues if the distance d_{ij} separating them is lower than 2 km, and the weight of each link is given by $\log(1/d_{ij})$ [32]. Figure S14 shows the partitions obtained on the network of commercial activities in Barcelona for $p = 0.5$ and $c = 1$ (**a**), $c = 2$ (**b**) and $c = 1000$ (**c**). For $c = 1$, the venues organize in spatial communities, determined solely by the relative distance among nodes. Some communities split already for $c = 2$, leading to a grouping of venues of the same type. Still, the more isolated spatial communities do not split until $c = 1000$, where most venues of the same category are clustered together. Whereas the results in Fig. S14 correspond to $p = 0.5$, by changing p we can also tune the typical size of the spatial communities, with higher values leading to smaller groups. For additional results using three other cities, see Figs. S7 and S8. We provide similar results on the spatial clusterisation for urban activities in Berlin (Fig. S15) and Prague (Fig. S16).

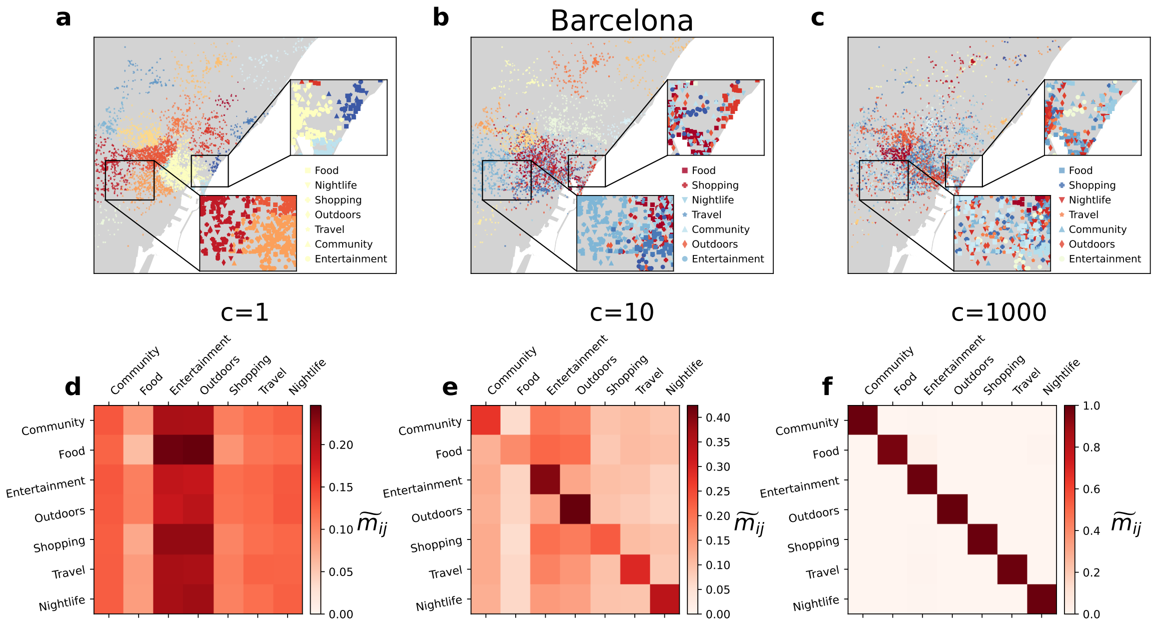

Figure S14: **Partitions obtained for the Gowalla venues in Barcelona.** The venues in Barcelona tracked by Gowalla user activity form a spatial graph where any pair of venues is connected by a link if they are less than 2 km apart. Nodes are divided into six classes, according to the type of venue. The partitions obtained for $p = 0.5$ and $c = 1$ (**a**), $c = 10$ (**b**) and $c = 1000$ (**c**) are reported. **d-f** Class overlapping $\widetilde{m}_{ij}$ when $c = 1$ (**d**), $c = 10$ (**e**) and $c = 1000$ (**f**). Venues are colored according to their community assignment and the marker indicates the type of venue.

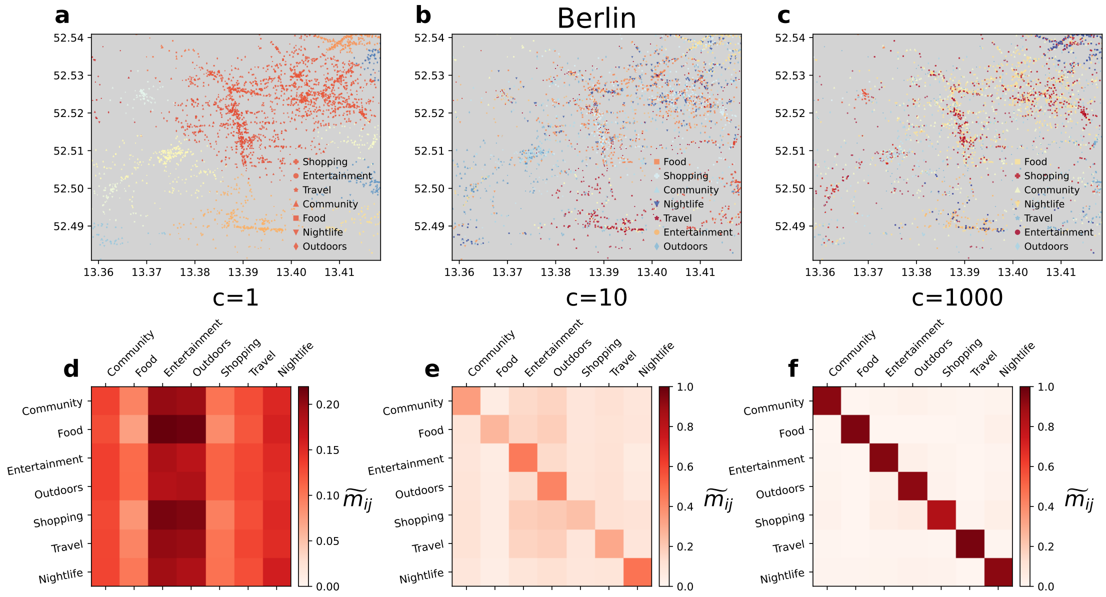

Figure S15: **Partitions obtained for the Gowalla venues in Berlin.** Community detection analysis in the city of Berlin on the spatial graph connecting any pair of venues if their are closer than 2 km. For a probability $p = 0.5$, partitions when $c = 1$ (**a**), $c = 10$ (**b**) and $c = 1000$ (**c**). **d-f** Class overlapping $\widetilde{m}_{ij}$ when $c = 1$ (**d**), $c = 10$ (**e**) and $c = 1000$ (**f**). Venues are colored according to their community assignment and the marker indicated the type of venue.

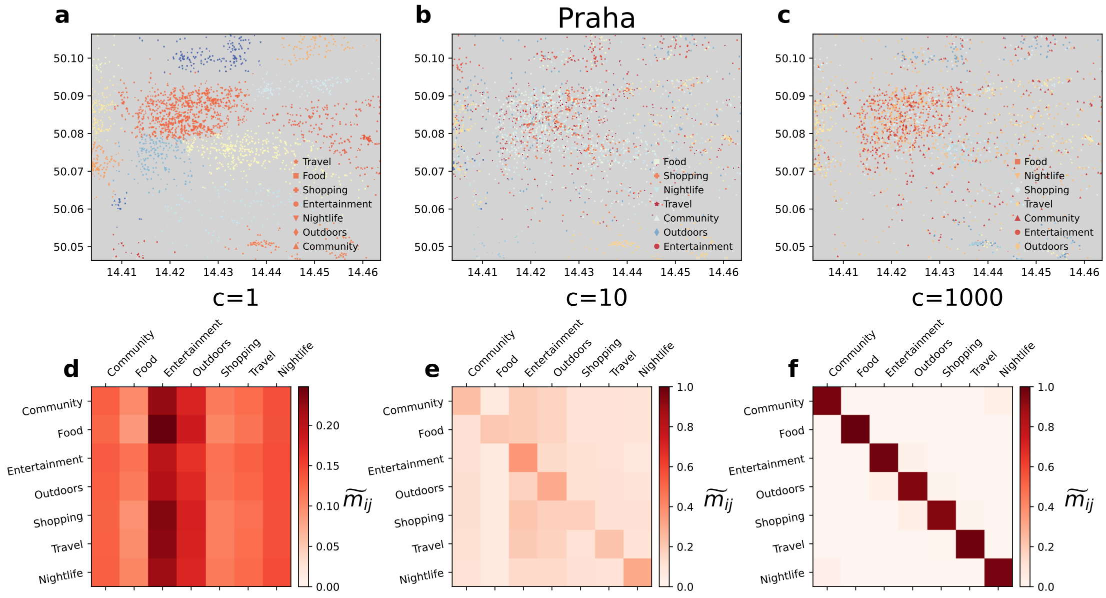

Figure S16: **Partitions obtained for the Gowalla venues in Prague.** Community detection analysis in the city of Prague on the spatial graph connecting any pair of venues if their are closer than 2 km. For a probability $p = 0.5$, partitions when $c = 1$ (**a**), $c = 10$ (**b**) and $c = 1000$ (**c**). **d-f** Class overlapping $\widetilde{m}_{ij}$ when $c = 1$ (**d**), $c = 10$ (**e**) and $c = 1000$ (**f**). Venues are colored according to their community assignment and the marker indicated the type of venue.
